# Are preventive measures adequate? An evaluation of the implementation of COVID-19 prevention and control measures in nursing homes in China

**DOI:** 10.1186/s12913-021-06690-z

**Published:** 2021-07-03

**Authors:** Meihong Shi, Fengying Zhang, Xinxin He, Siyuan Huang, Mingfeng Zhang, Xiuying Hu

**Affiliations:** 1 West China School of Nursing, Sichuan University/ Innovation Center of Nursing Research, West China Hospital, Sichuan University, PO Box 610041, Chengdu, Sichuan Province People’s Republic of China; 2 Nursing Department of Southwest Medical University, No. 1 Xianglin Rode, Longmatan District, PO Box 646000, Luzhou, Sichuan People’s Republic of China

**Keywords:** COVID-2019, Nursing homes, Prevention and control, Implementation, Evaluation, Transformational leadership, Prevention resource

## Abstract

**Background:**

The novel coronavirus disease 2019 (COVID-19) pandemic has become a challenge for nursing homes in China. Nursing homes are particularly dangerous places in terms of the spread of COVID-19 given that they house vulnerable, high-risk populations. As such, several useful guidelines for coping with COVID-19 in nursing homes have been provided. However, the actual implementation rates of such guidelines are unknown. This study aims to document the adherence of nursing homes to the Ministry of Civil Affairs guidelines for COVID-19 prevention and control in nursing homes.

**Methods:**

A cross-sectional study was conducted among 484 nursing homes in 136 cities of 28 provinces in China. A self-report questionnaire was created based on the Ministry of Civil Affairs guidelines for COVID-19 prevention and control in nursing homes (first edition). The questionnaire and the Transformational Leadership in the Public Sector Scale were sent to nursing home managers via the Wenjuanxing app online from February 7 to 29, 2020. Ultimately, 461 of 960 nursing homes participated, for a response rate of 48.0%.

**Results:**

The average overall implementation rate of COVID-19 prevention and control measures was 80.0% (143.97/180). The average implementation rates for hygienic behaviour management and access management were lower, at 75.3 and 78.7%, respectively. Number of medical staff and transformational leadership score of nursing home’s manager were associated with total implementation score (*p* < 0.05). A total of 69.8% (322/461) of the nursing home managers had serious resource problems, and inadequate protective supplies (72.0%) and staff shortages (47.7%) were the two primary problems. The nursing homes that located in urban, with large nursing home size, had hospital-nursing home cooperation and the transformational leadership score of manager> 60, had a lower risk of having serious resource problems.

**Conclusions:**

Overall, the implementation of prevention and control measures by nursing homes are insufficient during the epidemic in China. More medical staff, adequate resource, cooperation with hospitals, and higher transformational leadership of manager are required to improve the implementation rate. It is urgent for nursing homes to maintain the safety of residents and staff.

**Supplementary Information:**

The online version contains supplementary material available at 10.1186/s12913-021-06690-z.

## Background

The novel coronavirus disease 2019 (COVID-19) pandemic has affected the entire world [1], with more than 120.3 million confirmed cases and 2.6 million casualties by April 22, 2021 (World Health Organization, WHO). As the COVID-19 pandemic has continued, it has challenged the health system and impacted the lives of humans around the world. Given the absence of effective pharmaceutical interventions, non-pharmacological interventions (NPIs) are required to decrease disease transmission [2]. The adoption of non-pharmaceutical interventions such as mass confinement, social isolation, increased sanitation, and strict quarantine measures has proven to be beneficial in containing the virus [3].

Nursing homes are particularly dangerous places regarding the spread of COVID-19 given that they house vulnerable, high-risk populations. Older people are susceptible to novel coronavirus pneumonia [4, 5]. Approximately 12.6% of the nearly 55 million adults aged 65 and over suffer from respiratory illness in the USA [6]. This high prevalence of respiratory illness among the elderly population explains why COVID-19 is particularly lethal among this age group. Elderly residents of nursing homes are considered extremely vulnerable to COVID-19 exposure, infection, and disease consequences due to their high incidence of chronic disease and poor health in general. Furthermore, nursing homes provide an ideal environment for the spread of COVID-19 [7], as residents often live together in a crowded place, share sources of air and food, and share bedrooms and bathrooms. In the case of older vulnerable residents who usually require close care and therefore cannot completely adhere to social distancing guidelines both the residents and the workers around them are at high risk of becoming infected with COVID-19 [8]. An increasing number of reports have referenced the spread of COVID-19 among nursing homes in Hungary [7] and in Canada [9]. COVID-19 has also been documented in most nursing homes throughout the United States [10, 11]. As of May 28, 2020, 7500 American nursing homes reported 217,000 COVID-19 cases, and more than 44,000 coronavirus deaths had been reported in nursing and long-term care facilities, accounting for 50% of the country’s deaths to date [12]. 24% of staff in nursing homes were reported to have infected [13]. In European countries, such as Italy [14], Spain [15], and England [16], 40% ~ 57% of all COVID-19 deaths have occurred in nursing homes. Outbreaks in nursing homes can threaten the health care system [17], and the immediate implementation of prevention and control measures in nursing homes is needed. As such, several useful guidelines on coping with COVID-19 in nursing homes have been provided [18–20].

Approximately 4 million people reside in 31,997 nursing homes in China. Nursing home administrators faced challenges related to the potential spread of COVID-19 infections in nursing homes in late 2019. At the beginning of the COVID-19 pandemic, from managers to the staff had no plan for the pandemic. There was no definite treatment for COVID-19, and prevention was considered the best possible defence at their disposal at that moment. Guidance for nursing homes did not become available until January 28, 2020. The Ministry of Civil Affairs published the first edition of the guidelines for COVID-19 prevention and control in nursing homes, which provided prevention and control measured to address the urgent issue. In these guidelines, the minimum standards for nursing homes were published.

All of the nursing homes in China faced the threat of COVID-19 and implemented similar strategies following the guidance of the Ministry of Civil Affairs, but the extent of their preparedness, the swiftness with which the decisions were made and the scale of the measures varied. This paper focuses on adherence to guidelines for reducing virus transmission in nursing homes in China. We argue that there is an urgent need to evaluate the implementation rate of prevention and control measures in different nursing homes and analyse the factors related to their implementation.

## Methods

### Recruitment of nursing homes

This was a cross-sectional study conducted online. We searched for open-access information about nursing homes on the Civil Affairs websites of all provinces of China. We used a computerized random number generator to draw a sample of 480 nursing homes, which included approximately 1.5% of all nursing homes in China. Given the high decline rate in the online survey, we drew another sample of 480 nursing homes as reserve nursing homes in case institutions declined to participate. We called the nursing homes and described the goal of our survey and asked if they would be interested in participating in our survey. In the first phase, of the 480 invited nursing homes, 270 institutions declined to participate. In the second phase, of the remaining 480 nursing homes, 206 nursing homes declined. Finally, 484 nursing homes agreed to participate in our survey.

### Participants

Eligible participants were the managers of the 484 nursing homes. We added the managers as friends on the WeChat app, which is a social connection application widely used in China, and sent them the questionnaire through the app. The inclusion criteria were as follows: managers who were in charge of nursing homes, elderly care settings, or long-term care facilities in China and who agreed to fill out the questionnaire online. The exclusion criteria were a lack of knowledge about the nursing home’s actual situation and the completion of the questionnaire too quickly (in less than 120 s).

### Basic information

The basic information questions collection information on the demographic characteristics of managers, nursing home characteristics (Table 1). We also collected nursing homes’ problems during the pandemic, which was a multiple-choice question, including lack of daily necessities (food, consumables, and basic medicine), lack of protective supplies (face masks, gloves, alcohol, and disinfectant), lack of staff (medical staff, nurse aides, and other staff), difficulties with nursing home daily operation, lack of family comfort for the elderly residents, lack of psychological intervention for the elderly residents and staff, elderly residents’ problems seeing a doctor and other difficulties (Fig. 2). We defined a nursing home as one with serious resource problems when there was a combination of a lack of daily necessities, a lack of protective supplies and a lack of staff at the same time.

**Table 1:** Characteristics of the nursing homes and prevention and total implementation scores

Characteristic	Nursing homes		Total implementation score	*p*-value
	n	%	Median (P25, P75)	
Ownership				
Government-owned	93	20.2	145.0(117.5157.5)	0.011
Private-owned	287	62.3	151.0(133.0,162.0)	
Government-built, for-profit management	81	17.5	149.0(136.5162.5)	
Location				
Rural	75	16.3	145.0(113.0,159.0)	0.019
Urban	386	83.7	150.0(113.0,161.0)	
Nursing home size (number of beds)				
Small (< 100 beds)	149	32.3	147.0(123.0,159.0)	0.015
Medium (100–200 beds)	160	34.7	148.5(132.0,160.0)	
Large (more than 200 beds)	152	33.0	152.5(135.2164.0)	
Ratio of elderly residents to nurse aides				
> 15.00	50	10.8	139.0(112.5158.7)	0.044
10.01 ~ 15.00	34	7.4	148.5(125.5159.0)	
≤ 10.00	377	81.8	151.0(133.0,161.0)	
Presence of hospital-nursing home cooperation				
No	353	76.6	147.0(128.5160.0)	0.009
Yes	108	23.4	155.0(140.0,162.0)	
Number of medical staff				
None	91	19.7	137.0(112.0,158.0)	*P <* 0.001
1 ~ 5	199	43.2	148.0(131.0,160.0)	
6 ~ 10	52	11.3	154.0(135.7166.7)	
11 ~ 20	51	11.0	155.0(143.0,162.0)	
> 20	68	14.8	155.0(143.0,164.7)	
Establishment of quarantine room/unit				
None	70	15.2	148.0(129.0,157.0)	*P <* 0.001
Quarantine room	256	55.5	147.0(107.0,160.0)	
Quarantine unit	135	29.3	155.0(124.0,165.0)	
With serious resource problems				
without	139	30.2	154.0(141.0,164.0)	0.001
with	322	69.8	147.5(127.0,159.0)	
TFL score of nursing home’s manager				
< 50	156	33.8	133.0(113.2152.0)	< 0.001
50 ~ 60	93	20.2	149.0(127.0,160.0)	
> 60	212	46.0	156.0(145.0,164.0)	

*TFL* represents Transformational leadership

### Questionnaire

#### First phase: development of the questionnaire items

The questionnaire was titled “Questionnaire of implementation of the prevention and control of COVID-19 in nursing homes during the pandemic”, which developed for this study was provided as Additional file 1.Fifty-two items related to the implementation of prevention and control measures were created based on the Ministry of Civil Affairs guidelines for COVID prevention and control in nursing homes (first edition) [18]. We invited ten managers of nursing homes to read the items to help us make the questionnaire clearer.

#### Second phase: content validity and construct validity of the questionnaire items

We tested the questionnaire content validity with 14 experts (geriatric care specialists, psychologists, public health specialists, and nursing management specialists). The experts were required to rate the relevance of each item on a 4-point Likert scale from 1 (“completely irrelevant”) to 4 (“completely relevant”). The experts were asked to give revision suggestions for the questionnaire. We revised items with similar content or unclear expressions based on the suggestions of the experts. A cut-off of 0.78 for the item-level content validity index (I-CVI) was used for item retention [21]. After the removal of 13 irrelevant items, the scale-level content validity index (S-CVI) of the questionnaire was calculated. The content validity of the entire questionnaire was 0.810.

Exploratory factor analysis (EFA) was performed to estimate the construct validity. Total variance explained (%) was 57.79%, which satisfied the requirements for the Kaiser–Mayer–Olkin value (0.834) and a significant Bartlett’s test of sphericity (*p* < 0.001). These coefficients were all within the acceptable range [22]. Finally, 36 items were divided into the following four aspects based on factor analysis and professional judgment: basic management (7 items), access management (10 items), environmental disinfection management (7 items), and hygiene behaviour management (12 items).

#### Third phase: testing of the questionnaire

The questionnaire was sent to the managers of nursing homes. The managers were required to complete the questionnaire based on the nursing home’s actual situation. The managers were asked to indicate the frequency of the implementation of prevention and control measures in accordance with the recommendations of the Ministry of Civil Affairs in the nursing home where they worked at during the last week on a 5-point Likert scale (1 = never, 2 = rarely, 3 = sometimes, 4 = most of time, 5 = always). The total implementation score was the sum of all the scores of the 36 items. The higher the total implementation score was, the better the overall implementation of prevention and control in the nursing home. Average implementation rate = (every item mean÷ every item maximum) × 100%. The higher the average implementation rate of the item was, the better prevention and control implementation for this item.

We evaluated the questionnaire reliability after the completion of the first stage of the survey by analyzing 210 samples. We tested the internal consistency as a reliability value. Cronbach’s alpha of the questionnaire was 0.866. We tested the test-retest reliability of the questionnaire using the intraclass correlation coefficient (ICC) in ten nursing homes, with an interval of 2 weeks. The ICC was 0.822. These reliability values were acceptable [23].

### Scale

We used the scale “Transformational Leadership in the Public Sector Scale”, which was developed by Chinese author Wang Junxia in 2018 [24]. The scale was developed based on Bass and Avolio’s the theory of transformational leadership (TFL). Considering nursing homes’ social and public characteristics, we got the author’s permission to use this scale to explore the relationship between the transformational leadership of managers and the prevention and control implementation rate. The scale contains four dimensions: intellectual stimulation (4 items), inspirational motivation (2 items), individual care (3 items), and idealized influence (4 items). (see Additional file 2). The Cronbach’s alpha coefficient of the scale was reported by the scale developer to be 0.927. The items were scored using a 5-point Likert scale (1 = Strongly disagree to 5 = Strongly agree). Higher scores indicated higher levels of transformational leadership of nursing home managers.

### Data collection

The questionnaire and the Transformational Leadership in the Public Sector Scale were sent to the participants via the Wenjuanxing app, which is an e-questionnaire app. All questions in the questionnaire were required to be answered, and the questionnaire could not be submitted if it was not complete. The survey was conducted between February 7 and February 29, 2020, approximately 2 weeks after the beginning of the roll-out of the quarantine across China. Finally, we received 484 unique responses. After duplicates (from managers in the same nursing home or the same manager) and unqualified responses were removed, the final sample included 461 responses, with an analytic response rate of 48.0%.

### Data analysis

We used IBM SPSS Statistics (version 25.0, IBM Corporation) for data analysis. A descriptive analysis was performed by percentile. Continuous variables are presented median and interquartile range. The count data were expressed as the constituent ratio. Normality of distributions were tested using the Shapiro–Wilk test. The Wilcoxon test, Kruskal-Wallis test, univariate and multivariate binary logistic regression analysis were used for statistical analysis. Results are presented as odd ratio (OR) and 95% confidence interval (CI) in binary logistic regression analysis, *p* values < 0.05 were considered statistically significant.

## Results

### Characteristic of the nursing homes

The sample was 43.0% women (*n* = 198), and the average age was 43.0 years (Standard deviation = 10.46, range = 20–78 years). Managers were from 461 nursing homes in 134 cities in 28 provinces, and the average tenure in the current nursing home was 6.6 years (Standard deviation =5.4, range 1 to 30 years). A total of 4.3% of managers had a Master’s degree or above, 33.0% of managers had a Bachelor’s degree, 38.0% of managers had a technical college degree, 18.0% of managers had a high school diploma, and 6.7% had a junior high school diploma. There were 63,188 older adults living in the 461 nursing homes, who were cared for by 16,057 staff members (including managers, administrators, medical staff, nurse aides, and other staff members).

Table 1 showed the nursing home characteristics and total implementation score. A total of 19.7% of nursing homes did not have medical staff. Transformational leadership score of nursing home’s manager > 60 accounted for 46.0%. A total of 69.8% (322/461) of the nursing home managers reported that they had serious resource problems. Total implementation scores were found to be statistically significant difference in all the characteristics of nursing homes (*p* < 0.05) (Table 1).

### Implementation score and average implementation rate

The average implementation rate of COVID-19 prevention and control measures in the nursing homes was 80.0%. The managers reported a high average level of compliance with guidelines for the basic management of nursing homes, with an average implementation rate of up to 90.4%; the next highest average level of compliance was found for the environmental disinfection management aspect (79.0%). The average implementation rates for access management and hygiene behaviour management were the lowest, at 78.7 and 75.3%, respectively (Table 2).

**Table 2:** Evaluation implementation of prevention and control measures for COVID-19 in nursing homes

Items	Maximum	Mean	Average implementation rate (%)
**1 Basic management**	**35**	**31.64**	**90.4**
1.1 Implement home quarantine or in-nursing home quarantine for elderly residents who have left the nursing home	5	4.70	94.0
1.2 Monitor body temperature of residents and staff	5	4.07	81.4
1.3 Conduct bedroom patrol and daily active monitoring of symptoms.	5	4.52	90.4
1.4 Provide residents with emotional support and psychological counselling during the pandemic	5	4.07	81.4
1.5 Train staff to address COVID-19 and provide guidance for employees regarding the COVID-19 outbreak	5	4.69	93.8
1.6. Keep an eye on the epidemic	5	4.80	96.0
1.7 Report to the local public health authorities and follow the guidance	5	4.81	96.2
**2 Access management**	**50**	**39.35**	**78.7**
2.1 Notify elderly residents and family members of the suspension of all visitors to the nursing home	5	4.88	97.6
2.2 Prohibit any visitors unless it is for “an end-of-life situation”. Register the visitor and monitor the temperature of visitors, and require them to wear face masks and disinfect their hands	5	3.66	73.2
2.3 Arrange special reception rooms in the nursing home. Permit visitors in the reception room only under special circumstances, prohibit them from entering the living area	5	2.94	58.8
2.4 Offer staff accommodation in nursing homes	5	3.94	78.8
2.5 Perform centralized management of the accommodation site for staff	5	2.33	46.6
2.6 Require staff who enter the nursing home to wear face masks and disinfect their hands	5	3.24	64.8
2.7 Suspend all activities (consulting and reception services and unnecessary volunteer activities and social practices)	5	4.61	92.2
2.8 Suspend the acceptance of new elderly residents	5	4.66	93.2
2.9 Do not permit the residents to go out	5	4.75	95.0
2.10 Arrange for one staff member to receive daily necessities, ordered materials and other packages from family members and disinfect them with 75% alcohol or chlorine disinfectant before giving them to the elderly residents	5	4.32	86.4
**3 Environmental disinfection management**	**35**	**27.65**	**79.0**
3.1 Open windows for ventilation and improve air flow at least twice per day	5	4.35	87.0
3.2 Wipe the residents’ bedrooms with clean water at least twice per week	5	3.63	72.6
3.3 Disinfect the residents’ bedrooms with chlorine disinfectant at least twice per week	5	3.84	76.8
3.4 Wipe offices and service areas, including switches, elevator buttons, doorknobs, handrails, faucets, tables, and chairs, with clean water at least twice per week	5	3.52	70.4
3.5 Disinfect offices and service areas, including switches, elevator buttons, doorknobs, handrails, faucets, tables, and chairs, with chlorine disinfectant at least twice per week	5	3.77	75.4
3.6 Disinfect the kitchen, toilet, laundry, and garbage disposal areas with chlorine disinfectant at least once per day	5	3.94	78.8
3.7 Disinfect dining and drinking utensils at least three times per day	5	4.58	91.6
**4 Hygiene behaviour management**	**60**	**45.22**	**75.3**
4.1 Ask the residents to wash and disinfect their hands and maintain personal hygiene and help them do so	5	4.03	80.6
4.2 Ask elderly residents to close the toilet lid before flushing	5	3.40	68.0
4.3 Avoid eating together or communal meals, maintain physical distance, and suspend group activities	5	3.85	77.0
4.4 Provide knowledge education to the residents about COVID-19 prevention and control	5	3.11	62.2
4.5 Have staff wear face masks during working time	5	4.50	90.0
4.6 Have residents wear face masks in public areas of the nursing home	5	3.02	60.4
4.7 Have staff and residents wear face masks as appropriate and change into new face masks	5	2.96	59.2
4.8 Have staff wash their hands with soap and water or disinfect their hands using alcohol-based hand sanitizers before touching the residents	5	3.70	74.0
4.9 Have staff wash their hands with soap and water or disinfect their hands using alcohol-based hand sanitizers after touching the residents	5	3.78	75.6
4.10 Ensure that staff appropriately dispose of garbage, sewage and filth	5	4.13	82.6
4.11 Ensure that staff dispose or disinfect them face masks safely, without causing contamination.	5	4.13	82.6
4.12 Have staff implement administrative provisions related to food safety	5	4.52	90.4
**Total Implementation Score**	**180**	**143.97**	**80.0**

**Fig. 1:**
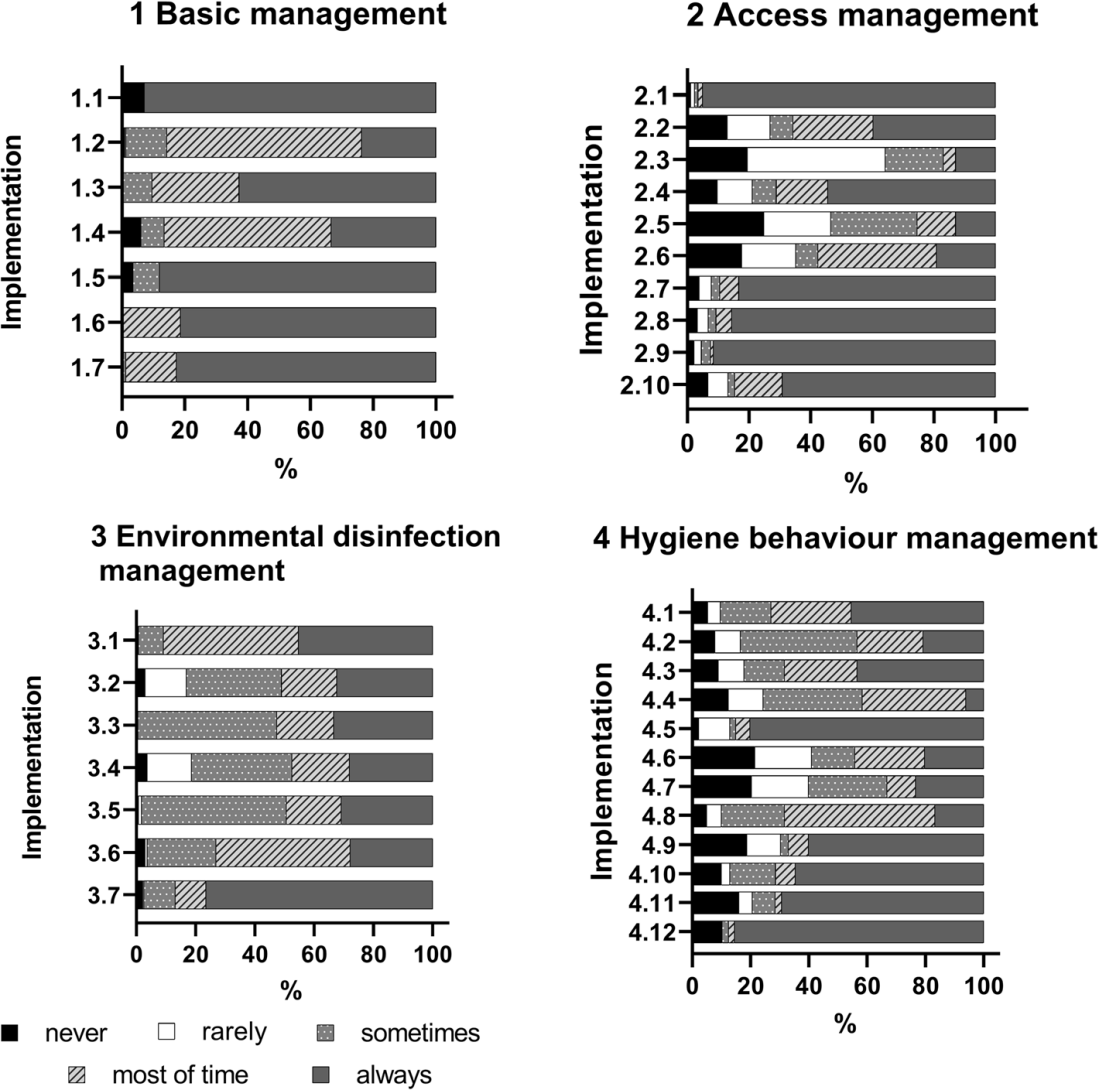
Implementation rates for the four aspects of COVID-19 prevention and control measures. Legend of Fig.1: A large proportion of nursing homes were unable to strictly adhere to the guidelines for some important items, for example, Item 2.3 and Item2.5, which were related to staff access management; Item2.2 and Item 2.6, which were related to visitor policy; Item 4.6,4.7,4.8, which were related to measures regarding wearing face masks; and Item 4.4, which was related to knowledge education to residents. Items’ detailed content is provided in Table 2

A large proportion of nursing homes were unable to strictly adhere to the guidelines for some important items, for example, Item 2.3 and Item2.5, which were related to staff access management; Item2.2 and Item 2.6, which were related to visitor policy; Item 4.6,4.7,4.8, which were related to measures regarding wearing face masks; and Item 4.4, which was related to knowledge education to residents (Fig. 1).

### Facility characteristics associated with total implementation scores

We used binary regression to analyze the facility characteristics associated with total implementation scores. Total implementation score used as tow-level independent variable. Y = 0 represents total implementation score < average, Y = 1 represents total implementation score ≥ average. The average of total implementation score was 143.97(Table 2). In adjusted multivariate model, number of medical staff and transformational leadership score of nursing home’s manager were persistently associated with total implementation score (*p* < 0.05). Nursing homes that, with less medical staff and a lower transformational leadership score of nursing homes manager, had a higher risk of get total implementation scores <average (Table 3).

**Table 3:** Facility characteristics associated with total implementation score

Characteristics	Total implementation score		Multivariate	
	<average (n, %)	≥average (n, %)	Adjusted OR	*p*-value
Ownership				
Government-owned	45(25.0)	48(17.1)	1.000	
Private-owned	103(57.2)	184(65.5)	2.341(0.808 ~ 6.780	0.117
ȃGovernment-built, for-profit management	32(17.8)	49(17.4)	1.903(0.604 ~ 5.991)	0.272
Location				
Rural	35(19.4)	40(14.2)	1.000	
Urban	145(80.6)	241(85.8)	0.4(0.122 ~ 1.317)	0.132
Nursing home size (number of beds)				
Small (< 100 beds)	62(34.4)	87(31.0)	1.000	
Medium (100 ~ 200 beds)	64(35.6)	906(34.2)	0.748(0.431 ~ 1.297)	0.301
Large (more than 200 beds)	54(30.0)	98(34.8)	0.620(0.317 ~ 1.211)	0.161
Ratio of elderly residents to nurse aides				
> 15.00	27(15.0)	23(8.2)	1.000	
10.01 ~ 15.00	14(7.8)	20(7.1)	1.343(0.510 ~ 3.534)	0.551
≤ 10.00	139(77.2)	238(847)	1.113(0.521 ~ 2.379)	0.781
Hospital-nursing home cooperation				
No cooperation	146(81.1)	207(73.7)	1.000	
Cooperation	34(18.9)	74(26.3)	0.926(0.517 ~ 1.658)	0.795
Number of medical staff				
None	51(28.3)	40(14.2)	1.000	
1 ~ 5	84(46.7)	115(40.9)	1.785 (0.947 ~ 3.367)	0.073
6 ~ 10	14(7.7)	38(13.6)	4.759 (1.903 ~ 11.899)	0.001
11 ~ 20	12(6.7)	39(13.9)	6.156 (2.074 ~ 18.269)	0.001
> 20	19(10.6)	49(17.4)	5.290 (1.688 ~ 16.582)	0.004
Establishment of quarantine room/unit				
None	28(15.6)	42(14.9)	1.000	
Quarantine room	109(60.6)	147(52.3)	0.669 (0.354 ~ 1.261)	0.214
Quarantine unit	43(23.8)	92(32.8)	0.899 (0.410 ~ 1.971)	0.791
With serious resource problems				
without	42(23.3)	97(34.5)	1.000	
with	137(76.7)	184(65.5)	0.742 (0.372 ~ 1.481)	0.397
TFL score of nursing home’s manager				
< 50	96(53.3)	60(21.4)	1.000	
50 ~ 60	39(21.7)	54(19.2)	2.189 (1.261 ~ 3.800)	0.005
> 60	45(25.0)	167(59.4)	6.072 (3.725 ~ 9.899)	< 0.001

*TFL* represents Transformational leadership

### Resource problems in the nursing homes

**Fig. 2:**
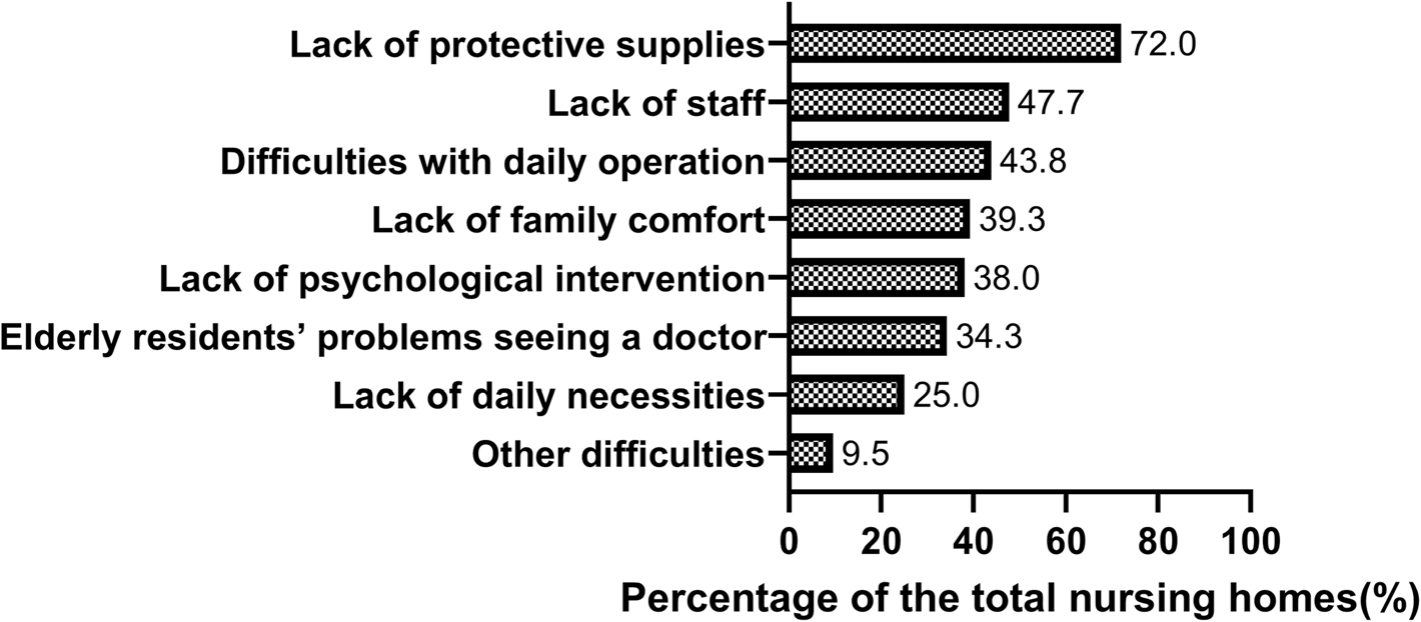
Resource problems of nursing homes in China. Legend of Fig.2: A total of 72.0% of the nursing homes reported a lack of protective supplies. The second problem of the nursing homes was staff shortages, with 47.7% of the nursing homes having this problem

We investigated the resource problems of nursing homes during the pandemic. The results showed that the primary need was protective supplies. A total of 72.0% of the nursing homes reported a lack of protective supplies. The second problem of the nursing homes was staff shortages, with 47.7% of the nursing homes having this problem (Fig. 2).

**Fig. 3:**
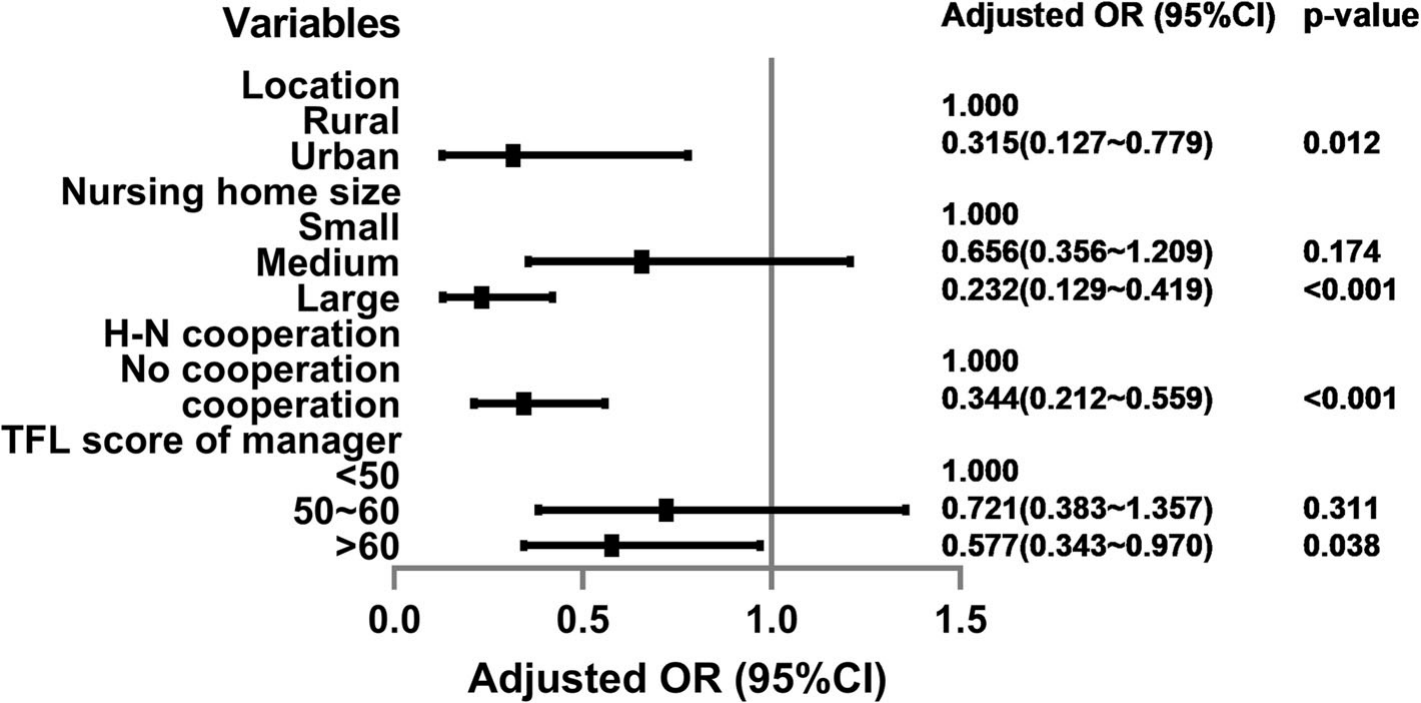
Factors related to nursing home with serious resource problems. Legend of Fig.3: The adjusted regression model showed that the nursing homes whose locations were in urban areas, with large nursing home size (more than 200 beds), having hospital-nursing home cooperation and the transformational leadership score of manager being above 60, had a lower risk of having serious resource problems. (Further details of the established model are given Supplementary material online, Additional file 3). H-N cooperation represents Hospital-Nursing homes cooperation; TFL represents Transformational leadership

We used multivariate binary logistic regression analysis to identify the characteristic of the nursing home with serious resource problems. When a nursing home had a combination of problems including a lack of daily necessities, a lack of protective supplies and a lack of staff at the same time, we defined it as nursing home with serious resource problem. The dependent variable Y = 0 represents nursing homes without serious resource problems, while Y = 1 represents nursing home with serious resource problems. We put characteristics of the nursing homes of Table 1 in the binary logistic regression analysis. Nursing home location, nursing home size, hospital-nursing home cooperation, and transformational leadership of manager were associated with nursing home serious resource problem (Fig. 3, Additional file 3).

## Discussion

### Medical support is a key point in helping nursing homes face the threat of COVID-19

The COVID-19 outbreak spread rapidly across the world. Nursing homes are high-risk settings and face unprecedented challenges. Various preventive measures were implemented in nursing homes in China, including lockdowns, the restriction of visitors, the prohibition of the entry of the people from Wuhan City, 14-day isolation for returning staff and residents, and symptom monitoring for all of the staff and residents. Despite these strict measures, COVID-19 still found its way into 3 nursing homes in Wuhan Province in China that were included in this survey. We must be aware of the many problems and weaknesses in the management of nursing homes. In this study, the average overall implementation rate of COVID-19 prevention and control measures was 80.0% (143.97/180), which was at the medium level. The staff and residents in nursing homes generally lacked awareness, knowledge, and basic skills for preventing the spread of infectious disease, which made the guidelines insufficient. The study shows that medical support and implementation rates were associated. This finding could be explained by the fact that less availability of resources, including medical human resources and less protective supplies, and poorly trained managers and staff, were related to lower medical support. Furthermore, lower medical support was associated with lower implementation rates.

In particular, the number of medical staff was a critical factor that affected the implementation score. This finding indicates that medical staff in nursing homes are poised to play a pivotal role in improving the implementation of COVID-19 prevention and control measures. These staff could provide services to residents directly, be responsible for operating the quarantine room/unit, and provide training to other staff. However, not all nursing homes had doctors and nurses on their staff. Research in the USA also showed a similar result: nursing homes with health care workers had a lower mortality rate than facilities without health care workers [25]. In our survey, there were no medical staff (doctors, nurses, or pharmacists) in 19.7% of the nursing homes, as nursing homes tend to strive to create a family environment rather than a medical environment [26]. This scenario leads to preventive measures that cannot be closely followed. Studies in Singapore [27] and Spain [15] suggest that public healthcare workers should be sent to nursing homes to help improve preparedness for and the prevention and control of COVID-19. A study in Canada also reported the experience of the emergency response of an acute-care hospital to a nursing home [28]. Such hospital-nursing home partnerships are a new form of cooperation that has arisen during the COVID-19 pandemic.

### A shortage of nurse aides in nursing homes was a significant problem

Nursing homes often face nurse aide shortages, which have been exacerbated by the current pandemic situation. Nurse aides have to work long shifts, perform high-intensity labour and complete a large amount of extra work during the pandemic because the nursing homes are experiencing high levels of absenteeism. The pandemic coincided with the Spring Festival. Some nurse aids who went home to reunite with their families were unable to return. Nurse aide shortages not only directly affect the achievement and maintenance of care quality in nursing homes but also indirectly affect the implementation of COVID-19 prevention and control measures [10]. Most of the implementation measures were implemented by the nurse aides. However, nearly half (47.7%) of the nursing homes reported staff shortages. Even before the pandemic, it was difficult to recruit qualified nurse aides in China. Nurse aides are often described as special groups with low social status, lower education level, insufficient training, and poor wages. Other countries face the same problem [27, 29, 30]. Policies should be designed to ensure that nursing homes are adequately staffed and that infection control protocols are implemented with high quality [17]. Common strategies include having nurse aides live in nursing homes, extending their hours, asking nurse aides to sacrifice their rest time and encouraging staff other than nurse aides to temporarily fill the nurse aide role. However, the execution of these strategies was difficult. It required a high level of leadership of the nursing home’s managers. The transformational leadership of the nursing home’ managers was different from other types of leadership. It built the relationship between managers and staff based on loyalty, trust and respect [31]. The transformational leadership was associated with the implementation of prevention and control measures in this survey. Managers elevated the moral level of staff through personal integrity [32]. Manager with higher transformational leadership encouraged staff to prolong the work hours, increase workload, and implement prevention and control measures to compensate for the shortage of staff.

### Little preparation for the pandemic

COVID-19 has exposed long-standing problems in nursing homes. Given the shockingly high rates of infections and deaths in nursing homes, it is crucial for nursing homes to prepare. However, little planning for infectious disease pandemics has been undertaken in nursing homes. There was no absolute requirement for such planning in the previous policy. Furthermore, in terms of the management system, there is a lack of emergency response plans, emergency leadership committees and emergency supply reserve mechanisms in nursing homes. This is similar to the situation in other countries, such as Ireland [33] and the USA [17], where there is little preparation for pandemics in nursing homes. Even more troubling is that most nursing homes lack the essential materials to protect their residents and staff. In our survey, 69.8% (322/461) of the nursing home managers reported that they had serious resource problems, and 72.0% of nursing homes reported that their primary problem was inadequate protective supplies. They could not obtain access to the necessary supplies for prevention at the beginning of the pandemic. The shortage of protective supplies is still a problem in many countries [34]. In total, 70% of American nursing homes are unable to provide sufficient supplies for their staff [10]. In this light, nursing homes should reserve protective material for emergency use, intensify their efforts to acquire protective supplies during epidemics, improve access to essential resources, and develop optimal distribution strategies for when supplies are insufficient. During the pandemic period, there may be considerable pressure on nursing homes to maintain operation; 43.8% of the nursing homes reported having difficulties operating, and 25.6% of nursing homes reported an inadequate supply of daily necessities in this study. Given the similar lack of preparation and reserves in the nursing home sector during many other natural disasters [33], the COVID-19 pandemic raises questions regarding whether nursing home managers have learned from this experience. We found that nursing homes those located in urban areas, those that had more than 200 beds, those that had hospital-nursing home cooperation, and those that managers with higher transformational leadership had a lower risk of serious resource problems. This finding indicates that the better a nursing home’s comprehensive conditions, the stronger its ability to resist the risk of resource problems caused by the COVID-19 epidemic. A similar result from nursing homes in the USA showed that nursing homes with higher quality ratings could control the spread of COVID-19 and decrease the death rate [35]. Both the human resources and material resources of the high-quality nursing homes were relatively sufficient [36]. Nursing homes in China must prepare enough human resources and material resources and make emergency response plans to avoid future crises.

### Access management should be strengthened

COVID-19 is an ongoing pandemic that is challenging nursing homes due to its high infectivity. It is forcing the implementation of drastic measures in nursing homes [37]. Nursing homes have adopted strict access and visitation restrictions according to the provided guidelines [38]. Overall, the rate of adherence to the guidelines for access management among nursing homes in this study was 78.7%. However, it is uncertain whether the level of adherence can be maintained throughout the pandemic period. Staff are most likely to cause imported transmission [39]. Staff movements between their homes and nursing home facilities could transmit the disease. A survey of Chinese nurse aides showed that 81% of nurse aides from a rural area had a low educational level and low adaption of other work. Ninety-two percent of nurse aides were inter-city migrants, and 62% of nurse aides were inter-provincial migrants. Unlike in other countries, Chinese nursed aides always work for only one facility and do not have a second job. Therefore, 85% of nursing homes accommodated nurse aides [40]. However, in our survey, only 78.8% of nursing homes could provide staff accommodations in nursing homes. To decrease the risk of the spread of COVID-19, one infection control measure is to house the staff in the nursing homes, which might mitigate the risk associated with staff moving between their homes and the facilities during the pandemic. If there is no living space in the nursing home, the staff should be centrally managed in an accommodation site without having contact with others. The staff responsible for purchasing should not contact staff who provide services to the residents. Access management is the key form of management; if not strictly implemented, nursing homes will be placed at great risk.

During the COVID-19 epidemic, most nursing homes in China were completely closed and had little contact with the external environment, with nobody entering and nobody exiting. In the early stage of the epidemic, nursing homes prohibited any visitors unless it was for “an end-of-life situation.” Item 2.2 and Item 2.3 were related to strict visiting policies. However, the average implementation rates for the measures described in Item 2.2 and Item 2.3 were not high, at 73.2 and 58.8%, respectively.

Lockdown and isolation are appropriate policies to limit the spread of COVID-19, but these measures impact people’s mental health [3]. Some residents and staff often feel lonely, anxious and depressed, and they struggle with the absence of relatives. In our study, 38.0% of elderly residents and staff needed psychological intervention during the lockdown period. It was unclear how long isolation would last by the time we finished our survey, but the situations may worsen as enforced isolation continues. Strict lockdown policies made family members and other volunteers’ support care impossible, further exposing the vulnerabilities associated with staff shortages [10]. Manager leadership might play critical role in maintaining the stability of nursing homes. Transformational leaders change their staff and encouraged them to consider organizational goals over personal interests [41]. Such leadership make staff regard fighting COVID-19 as a battle and make them view staying at the nursing home as heroic behaviour to guard the safety of the nursing home.

### Problems with environmental disinfection management

Environmental cleaning and disinfection are important precautionary measures to prevent indirect COVID-19 infections. Kampf et al. [42] reported that coronaviruses can live on surfaces for up to 9 days. Adopting environmental cleaning and disinfection measures has proven to be beneficial in containing the virus [43]. Therefore, the regular cleaning and disinfection of surfaces and objects are essential to control spread. However, the implementation rate of environmental disinfection management was only 79.0% in this study. The frequency of the cleaning and disinfection of residents’ bedrooms and office areas and service places was insufficient (Item 3.2 to 3.5). Although most of the nursing homes had established infection disease prevention and control standards, the staff were insufficiently trained in infectious disease prevention and control skills. The local CDC should be responsible for providing training to the staff in nursing homes regarding disinfection knowledge, including information about what kinds of disinfectants are effective and how to disinfection can be performed effectively [44]. Sanitation and environmental disinfection should be carried out scientifically based on the recommended procedures [45]. Enhanced environmental cleaning and disinfection in nursing homes is important; it should not be a temporary task but should be routine work.

### Risk in hygienic behaviour management

Hygienic behaviour management is central to the transmission of COVID-19, and changing behaviour is crucial to prevent transmission. In our survey, the average implementation rate of hygienic behaviour management was only 75.3%, which was the lowest rate among the four aspects of the implementation of COVID-19 prevention and control measures, indicating that hygienic behaviour management is the weakest point in nursing homes. In addition, the implementation rates of measures involving residents, such as having them wear face masks, providing them with education and maintaining physical distance, were extremely low, which may be related to the cognitive decline of some residents. Aktinson et al. [46] reported that the size of respiratory droplets from humans typically ranges from 0.5 to 12 μm and that droplets sized < 0.5 μm can remain airborne for significant periods of time. Van Doremalen et al. [47] found that COVID-19 remains stable in airborne aerosols for at least 3 h and can persist on inanimate surfaces for 48 to 72 h. Based on the above, personal protection in the form of improved hygiene behaviour [44], such as the appropriate utilization of face masks in public areas, may mitigate future COVID-19 transmission in nursing homes. Among the many considerations, 42 % of the elderly have dementia or cognitive decline in nursing homes [48], making the enforcement of mitigation strategies such as face mask wearing a major challenge [12]. Therefore, items related to wearing face masks as appropriate (Item 4.6 to 4.8) showed lower average implementation rates. It has been a controversial issue whether residents must wear masks in nursing homes that are already completely closed. Given that elderly residents often have respiratory diseases [6], wearing face masks will affect their respiratory function, which can lead to decreased compliance.

Effective risk communication and knowledge education in the early stage of the COVID-19 outbreak is critical for promoting behavioural compliance. These strategies have proven to be useful for the general public [3] but have not been effectively adopted in nursing homes. The implementation rate for the item “Provide knowledge education to the residents about COVID-19 prevention and control” (Item 4.4) was also very low (64.0%). For example, understanding why face masks should be used, why visitors are not coming to see them, and why they should keep distance from others is difficult for residents with dementia. It is also a major challenge for staff to provide education to these residents. The recommendation to “maintain physical distance” (Item 4.3) was also difficult for most elderly individuals and staff. The implementation rate for this measure was 77.0%. In China, residents who live in nursing homes have chronic underlying medical disorders; they usually have considerable personal care needs, including bathing, dressing, and toileting, which make maintaining physical distancing nearly impossible. Considering the characteristics of the residents, there are few strategies that can be used to improve the hygiene behaviour of residents. The best strategy is to prevent COVID-19 from invading nursing homes.

## Conclusions

The COVID-19 pandemic is perhaps one of the greatest health threats the world has faced in this century, and it requires not only a cohesive effort but also enormous discipline to follow the guidelines. The current implementation of prevention and control measures is insufficient, and further improvement is required. We argue that effective interventions are urgently needed to increase the implementation rate of access management, environmental disinfection management, and hygienic behaviour management. Shortages in medical staff and nurse aides, a lack of preventive supplies, and little preparation for pandemics are common obstacles encountered by nursing homes. Currently, prevention and control work still being undertaken in all nursing homes to ensure a high level of preparedness to combat COVID-19 in China. Local officials must inspect nursing homes, provide medical support and other helps; they must ensure that nursing homes are adequately staffed, that the residents’ needs are being met and that infection control procedures are being followed.

### Study considerations

There were some limitations to this study. The survey was conducted online; although we used the random sampling method, more than half of the contacted nursing homes refused to participate in the survey. The results may have been affected by selection bias because of the high decline rate. Because of the limitation of resources, we could not investigate a sufficient number of nursing homes. The number of nursing homes investigated was 461, which was less than the ideal sample size of 490. These results offer valuable insight into the nursing homes’ adherence to the guidelines. The implementation of prevention and control measures in nursing homes was evaluated from managers’ perspectives; therefore, caution should be exercised in generalizing these findings. Despite these limitations, to the best of our knowledge, this is the first study to evaluate the implementation of prevention and control measures in nursing homes during the early phase of the COVID-19 outbreak in China.

## Supplementary Information

**Additional file 1.** Questionnaire of implementation of the prevention and control of COVID-19 in nursing homes during the pandemic.

**Additional file 2.** Result of transformational leadership of nursing home’s manager.

**Additional file 3.** Binary logistic regression analysis of factors related to nursing home with serious resource problems.

## Data Availability

Questionnaire of implementation of the prevention and control of COVID-19 in nursing homes during the pandemic is provided as Additional files 1. Availability of data of Transformational leadership of nursing home’s managers was in Additional file 2. Binary logistic regression analysis of factors related to nursing home with serious resource problems was in Additional file 3.The datasets used and/or analysed during the current study available from the corresponding author on reasonable request.

## Abbreviations

COVID-19: The novel coronavirus disease 2019
WHO: World Health Organization
I-CVI: Item-level Content Validity Index
S-CVI: Scale-Level Content Validity Index
EFA: Exploratory Factor Analysis
ICC: Intraclass Correlation Coefficient
TFL: Transformational Leadership
OR: Odd Ratio
CI: Confidence Interval
H-N cooperation: Hospital-Nursing homes cooperation
