# Supplementary material for: Are preventive measures adequate? An evaluation of the implementation of COVID-19 prevention and control measures in nursing homes in China
Source: BMC Health Serv Res. 2021 Jul 3;21:641. doi: 10.1186/s12913-021-06690-z (PMC8254064; doi:10.1186/s12913-021-06690-z)
Supplement: Supplementary file 1 — Additional file 1. Questionnaire of implementation of the prevention and control of COVID-19 in nursing homes during the pandemic. [file 12913_2021_6690_MOESM1_ESM.docx]

Additional file 1 Questionnaire of implementation of the prevention and control of COVID-19 in nursing homes during the pandemic

Dear managers:

We are the research team of Sichuan University. In order to against the COVID-19 epidemic, we need to evaluation of the implementation of prevention and control and the demand of the nursing homes. We will keep your personal information confidential. Please feel free to fill in this questionnaire. Your answer is very important to our survey.

Thank you for your support and participation.

1. Would you like to take part in this survey [Single-choice topic] *

| ○Yes |
| --- |
| ○No |

2. Your gender [Single-choice topic] *

| ○Male |
| --- |
| ○Female |

3. Your age [fill in the blank] *

_________________________________

4. Your educational level [Single-choice topic] *

| ○Primary and below |
| --- |
| ○Junior high school diploma |
| ○High school diploma |
| ○College’s degree |
| ○Bachelor’s degree |
| ○Master degree or above |

5.How many years have you worked as manager in the nursing home（fill in numbers） [fill in the blank] *

_________________________________

6. Name of the nursing home [fill in the blank] *

_________________________________

7. City where is the nursing home [fill in the blank] *

_________________________________

8. Where does the nursing home locate? [Single-choice topic] *

| ○ urban |
| --- |
| ○ rural |

9. How many beds are there in the nursing home（fill in numbers） [fill in the blank] *

_________________________________

10. How many residents are currently housed in the nursing home（fill in numbers） [fill in the blank] *

_________________________________

11. Ownership of the nursing home [Single-choice topic] *

| ○Government-owned |
| --- |
| ○Private-owned |
| ○Government build for-profit management |
| ○ohters_________________ |

12. Is the nursing home as hospital-nursing home cooperation? [Single-choice topic] *

| ○Yes |
| --- |
| ○No |

13. In your nursing home, how many doctors ：_______，nurses：________，other medical staff_______，nurse aides：__________（Please enter a number or zero if none） [fill in the blank] *

14. Is there any quarantine room( unit) in your nursing home [Single-choice topic] *

| ○None |
| --- |
| ○Quarantine room |
| ○Quarantine unit |

15. Are there any confirmed and suspected COVID-19 cases in your nursing home？ [Single-choice topic] *

| ○Yes |
| --- |
| ○No |

|  | Items | never | rarely | sometimes | most of time | always |
| --- | --- | --- | --- | --- | --- | --- |
| 16 Basic management | |  |  |  |  |  |
|  | 1.1 Implement home quarantine or in-nursing home quarantine for elderly residents who have left the nursing home | ○ | ○ | ○ | ○ | ○ |
|  | 1.2 Monitor body temperature of residents and staff | ○ | ○ | ○ | ○ | ○ |
|  | 1.3 Conduct bedroom patrol and daily active monitoring of symptoms. | ○ | ○ | ○ | ○ | ○ |
|  | 1.4 Provide residents with emotional support and psychological counselling during the pandemic | ○ | ○ | ○ | ○ | ○ |
|  | 1.5 Train staff to address COVID-19 and provide guidance for employees regarding the COVID-19 outbreak | ○ | ○ | ○ | ○ | ○ |
|  | 1.6. Keep an eye on the epidemic | ○ | ○ | ○ | ○ | ○ |
|  | 1.7 Report to the local public health authorities and follow the guidance | ○ | ○ | ○ | ○ | ○ |
| 17 Access management | |  |  |  |  |  |
|  | 2.1 Notify elderly residents and family members of the suspension of all visitors to the nursing home | ○ | ○ | ○ | ○ | ○ |
|  | 2.2 Prohibit any visitors unless it is for “an end-of-life situation”. Register the visitor and monitor the temperature of visitors, and require them to wear face masks and disinfect their hands | ○ | ○ | ○ | ○ | ○ |
|  | 2.3 Arrange special reception rooms in the nursing home. Permit visitors in the reception room only under special circumstances, prohibit them from entering the living area | ○ | ○ | ○ | ○ | ○ |
|  | 2.4 Offer staff accommodation in nursing homes | ○ | ○ | ○ | ○ | ○ |
|  | 2.5 Perform centralized management of the accommodation site for staff | ○ | ○ | ○ | ○ | ○ |
|  | 2.6 Require staff who enter the nursing home to wear face masks and disinfect their hands | ○ | ○ | ○ | ○ | ○ |
|  | 2.7 Suspend all activities (consulting and reception services and unnecessary volunteer activities and social practices) | ○ | ○ | ○ | ○ | ○ |
|  | 2.8 Suspend the acceptance of new elderly residents | ○ | ○ | ○ | ○ | ○ |
|  | 2.9 Do not permit the residents to go out | ○ | ○ | ○ | ○ | ○ |
|  | 2.10 Arrange for one staff member to receive daily necessities, ordered materials and other packages from family members and disinfect them with 75% alcohol or chlorine disinfectant before giving them to the elderly residents | ○ | ○ | ○ | ○ | ○ |
| 18 Environmental disinfection management | |  |  |  |  |  |
|  | 3.1 Open windows for ventilation and improve air flow at least twice per day | ○ | ○ | ○ | ○ | ○ |
|  | 3.2 Wipe the residents’ bedrooms with clean water at least twice per week | ○ | ○ | ○ | ○ | ○ |
|  | 3.3 Disinfect the residents’ bedrooms with chlorine disinfectant at least twice per week | ○ | ○ | ○ | ○ | ○ |
|  | 3.4 Wipe offices and service areas, including switches, elevator buttons, doorknobs, handrails, faucets, tables, and chairs, with clean water at least twice per week | ○ | ○ | ○ | ○ | ○ |
|  | 3.5 Disinfect offices and service areas, including switches, elevator buttons, doorknobs, handrails, faucets, tables, and chairs, with chlorine disinfectant at least twice per week | ○ | ○ | ○ | ○ | ○ |
|  | 3.6 Disinfect the kitchen, toilet, laundry, and garbage disposal areas with chlorine disinfectant at least once per day | ○ | ○ | ○ | ○ | ○ |
|  | 3.7 Disinfect dining and drinking utensils at least three times per day | ○ | ○ | ○ | ○ | ○ |
| 19 Hygiene behavior Management | |  |  |  |  |  |
|  | 4.1 Ask the residents to wash and disinfect their hands and maintain personal hygiene and help them do so | ○ | ○ | ○ | ○ | ○ |
|  | 4.2 Ask elderly residents to close the toilet lid before flushing | ○ | ○ | ○ | ○ | ○ |
|  | 4.3 Avoid eating together or communal meals, maintain physical distance, and suspend group activities | ○ | ○ | ○ | ○ | ○ |
|  | 4.4 Provide knowledge education to the residents about COVID-19 prevention and control | ○ | ○ | ○ | ○ | ○ |
|  | 4.5 Have staff wear face masks during working time | ○ | ○ | ○ | ○ | ○ |
|  | 4.6 Have residents wear face masks in public areas of the nursing home | ○ | ○ | ○ | ○ | ○ |
|  | 4.7 Have staff and residents wear face masks as appropriate and change into new face masks | ○ | ○ | ○ | ○ | ○ |
|  | 4.8 Have staff wash their hands with soap and water or disinfect their hands using alcohol-based hand sanitizers before touching the residents | ○ | ○ | ○ | ○ | ○ |
|  | 4.9 Have staff wash their hands with soap and water or disinfect their hands using alcohol-based hand sanitizers after touching the residents | ○ | ○ | ○ | ○ | ○ |
|  | 4.10 Ensure that staff appropriately dispose of garbage, sewage and filth | ○ | ○ | ○ | ○ | ○ |
|  | 4.11 Ensure that staff dispose or disinfect them face masks safely, without causing contamination. | ○ | ○ | ○ | ○ | ○ |
|  | 4.12 Have staff implement administrative provisions related to food safety | ○ | ○ | ○ | ○ | ○ |

20. Problems and demand of nursing homes during the pandemic [Multiple choice topic] *

| □Inadequate supply of daily necessities (food, consumables, and basic medicine) |
| --- |
| □Inadequate protective supplies (face masks, gloves, alcohol, disinfectant, and PPE (personal protective equipment) |
| □The elderly had mental problems |
| □The elderly and staff need psychological intervention |
| □The absence of support from the elderly’s family member |
| □Staff shortages |
| □Lack of knowledge on epidemic prevention and control |
| □ The elderly have difficulty seeing a doctor |
| □The nursing homes are in the trouble of the operation |
| □ Others |

21. Transformational Leadershipin the Public Sector Scale

| Items | Strongly disagree | disagree | Sometimes  agree | agree | Strongly agree |
| --- | --- | --- | --- | --- | --- |
| I will encourage staff to explore new and better way to do things |  |  |  |  |  |
| I will empower employees to participate in important decisions | ○ | ○ | ○ | ○ | ○ |
| I will reward creativity and innovation | ○ | ○ | ○ | ○ | ○ |
| I encourage staff to look at things critically | ○ | ○ | ○ | ○ | ○ |
| I will make the staff feel that they have been given the appropriate power | ○ | ○ | ○ | ○ | ○ |
| I make the staff feel like they are family members of nursing homes | ○ | ○ | ○ | ○ | ○ |
| I will let the staff to balance at family and at work | ○ | ○ | ○ | ○ | ○ |
| I will give staff enough opportunity to use their talents | ○ | ○ | ○ | ○ | ○ |
| I will give staff a chance to show their leadership |  |  |  |  |  |
| I have always maintained a high level of honesty and integrity | ○ | ○ | ○ | ○ | ○ |
| I can fairly resolve grievances and disputes within the organization | ○ | ○ | ○ | ○ | ○ |
| I have been courageous in the pursuit of truth | ○ | ○ | ○ | ○ | ○ |
| I have always had a genuine desire to serve the people | ○ | ○ | ○ | ○ | ○ |

22. What is the greatest need for your nursing home during the epidemic [fill in the blank]

_________________________________
