# Supplementary material for: Are preventive measures adequate? An evaluation of the implementation of COVID-19 prevention and control measures in nursing homes in China
Source: BMC Health Serv Res. 2021 Jul 3;21:641. doi: 10.1186/s12913-021-06690-z (PMC8254064; doi:10.1186/s12913-021-06690-z)
Supplement: Supplementary file 2 — Additional file 2. Result of transformational leadership of nursing home’s manager. [file 12913_2021_6690_MOESM2_ESM.docx]

Additional file 2 Transformational leadership of nursing home’s manager

| Que | Items | Maximum | Mean |
| --- | --- | --- | --- |
| **Intellectual stimulation** | | **20** | **16.78** |
| Q1 | I will encourage staff to explore new and better way to do things | 5 | 4.49 |
| Q2 | I will empower employees to participate in important decisions | 5 | 4.11 |
| Q3 | I will reward creativity and innovation | 5 | 4.44 |
| Q4 | I encourage staff to look at things critically | 5 | 3.75 |
| **Inspirational motivation** | | **10** | **8.48** |
| Q5 | I will make the staff feel that they have been given the appropriate power | 5 | 4.14 |
| Q6 | I make the staff feel like they are family members of nursing homes | 5 | 4.34 |
| **Individualized consideration** | | **15** | **12.89** |
| Q7 | I will let the staff to balance at family and at work | 5 | 4.24 |
| Q8 | I will give staff enough opportunity to use their talents | 5 | 4.38 |
| Q9 | I will give staff a chance to show their leadership | 5 | 4.28 |
| **Idealized influence** | | **20** | **17.88** |
| Q10 | I have always maintained a high level of honesty and integrity | 5 | 4.48 |
| Q11 | I can fairly resolve grievances and disputes within the organization | 5 | 4.41 |
| Q12 | I have been courageous in the pursuit of truth | 5 | 4.44 |
| Q13 | I have always had a genuine desire to serve the people | 5 | 4.55 |
| **Total score of transformational leadership** | | **65** | **56.03** |

note: The average leadership score of the nursing home’s manager was 56.03. The score of four dimensions: intellectual stimulation (16.78), inspirational motivation (8.48), individual care (12.89), and idealized influence (17.88).
