# Supplementary material for: Are preventive measures adequate? An evaluation of the implementation of COVID-19 prevention and control measures in nursing homes in China
Source: BMC Health Serv Res. 2021 Jul 3;21:641. doi: 10.1186/s12913-021-06690-z (PMC8254064; doi:10.1186/s12913-021-06690-z)
Supplement: Supplementary file 3 — Additional file 3. Binary logistic regression analysis of factors related to nursing home with serious resource problems. [file 12913_2021_6690_MOESM3_ESM.docx]

Additional file 3 Binary logistic regression analysis of factors related to nursing home with serious resource problems

|  | Nursing home with serious resource problem | | | Univariate | | Multivariate | |
| --- | --- | --- | --- | --- | --- | --- | --- |
| Characteristics | Without | with |  | Crude OR | p-value | Adjusted OR | p-value |
| Ownership |  |  |  |  |  |  |  |
| Government-owned | 13(9.4) | 80(24.8) |  | 1.000 |  |  |  |
| Private-owned | 94(67.6) | 193(59.9) |  | 0.334(0.177~0.630) | 0.001 |  |  |
| Government-built, for-profit management | 32(23.0) | 49(15.3) |  | 0.249(0.119~0.520) | <0.001 |  |  |
| Location |  |  |  |  |  |  |  |
| Rural | 6(4.3) | 69(21.4) |  | 1.000 |  | 1.000 |  |
| Urban | 133(95.7) | 253(78.6) |  | 0.165(0.070~0.391) | <0.001 | 0.315(0.127~0.779) | 0.012 |
| Nursing home size (number of beds) | | |  |  |  |  |  |
| Small (<100 beds) | 21(15.1) | 128(39.8) |  | 1.000 |  | 1.000 |  |
| Medium (100~200 beds) | 39(28.1) | 121(37.6) |  | 0.509(0.293~0.914) | 0.024 | 0.656(0.356~1.209) | 0.174 |
| Large (more than 200 beds) | 79(56.8) | 73(22.6) |  | 0.152(0.087~0.266) | <0.001 | 0.232(0.129~0.419) | <0.001 |
| Ratio of elderly residents to nurse aides | | |  |  |  |  |  |
| >15.00 | 2(1.4) | 48(14.9) |  | 1.000 |  |  |  |
| 10.01~15.00 | 0(0.0) | 34(10.6) |  | 67311451.79(0.000) | 0.998 |  |  |
| $\leq$10.00 | 137(98.6) | 240(74.5) |  | 0.073(0.017~0.305) | <0.001 |  |  |
| Hospital-nursing home cooperation | | |  |  |  |  |  |
| No cooperation | 80(57.6) | 273(84.8) |  | 1.000 |  | 1.000 |  |
| Cooperation | 59(42.4) | 49(15.2) |  | 0.243(0.155~0.383) | <0.001 | 0.344(0.212~0.559) | <0.001 |
| Number of medical staff |  |  |  |  |  |  |  |
| None | 1(0.7) | 90(28.0) |  | 0.068(0.009~0.507) | 0.009 |  |  |
| 1~5 | 28(20.1) | 171(53.1) |  | 0.023(0.003~0.178) | <0.001 |  |  |
| 6~10 | 17(12.2) | 35(10.9) |  | 0.006(0.001~0.043) | <0.001 |  |  |
| 11~20 | 34(24.5) | 17(5.2) |  | 0.002(0.000~0.014) | <0.001 |  |  |
| >20 | 59(42.45) | 9(2.8) |  |  |  |  |  |
| Establishment of quarantine room/unit | | |  |  |  |  |  |
| None | 4(2.9) | 66(20.5) |  | 1.000 |  |  |  |
| Quarantine room | 52(37.4) | 204(63.4) |  | 0.172(0.115~0.256) | <0.001 |  |  |
| Quarantine unit | 83(59.7) | 52(16.1) |  |  |  |  |  |
| TFL score of nursing home’s manager | | |  |  |  |  |  |
| <50 | 34(24.5) | 122(37.9) |  | 1.000 |  | 1.000 |  |
| 50~60 | 30(21.5) | 63(19.6) |  | 0.585(0.329~1.043) | 0.069 | 0.721(0.383~1.357) | 0.311 |
| >60 | 75(54.0) | 137(42.5) |  | 0.509(0.317~0.817) | 0.005 | 0.577(0.343~0.970) | 0.038 |
| Total | 139(30.2) | 322(100.0) |  |  |  |  |  |

note: The variables included ownership (X1=1 represents Government-owned; X1=2 represents Private-owned; X1=3 represents Government-built, for-profit management.), location (X2=1 represents Rural; X2=2 represents Urban), nursing home size (X3=1represents Small (<100 beds); X3=2 represents Medium (100-200 beds); X3=3 represents Large (more than 200 beds), ratio of elderly resident to nurse aides (4=1 represents Ratio of elderly residents to nurse aides >15.00; X4=2 represents >15.00; X4=3 represents10.01~15.00; X4=4 represents$\leq$10.00), Hospital-nursing home cooperation (X5=0 represents No cooperation; X5=1 represents cooperation), number of medical staff (X6=0 represents None; X6=1 represents 1~5; X6=2 represents 6~10; X6=3 represents 11~20; X6=4 represents >20), establishment of quarantine room/unit (X7=0 represents None; X7=1 represents Quarantine room; X7=2 represents Quarantine unit.), and TFL score of nursing home’s manager (X8=1 represent <50; X8=2 represents 50~60; X8=3 represents >60). TFL represents Transformational leadership.
